# Meta-Analyzing Multiple Omics Data With Robust Variable Selection

**DOI:** 10.3389/fgene.2021.656826

**Published:** 2021-07-05

**Authors:** Zongliang Hu, Yan Zhou, Tiejun Tong

**Affiliations:** ^1^College of Mathematics and Statistics, Shenzhen University, Shenzhen, China; ^2^Department of Mathematics, Hong Kong Baptist University, Kowloon Tong, Hong Kong

**Keywords:** heterogeneity, logistic regression, meta-analysis, robust estimation, variable selection

## Abstract

High-throughput omics data are becoming more and more popular in various areas of science. Given that many publicly available datasets address the same questions, researchers have applied meta-analysis to synthesize multiple datasets to achieve more reliable results for model estimation and prediction. Due to the high dimensionality of omics data, it is also desirable to incorporate variable selection into meta-analysis. Existing meta-analyzing variable selection methods are often sensitive to the presence of outliers, and may lead to missed detections of relevant covariates, especially for lasso-type penalties. In this paper, we develop a robust variable selection algorithm for meta-analyzing high-dimensional datasets based on logistic regression. We first search an outlier-free subset from each dataset by borrowing information across the datasets with repeatedly use of the least trimmed squared estimates for the logistic model and together with a hierarchical bi-level variable selection technique. We then refine a reweighting step to further improve the efficiency after obtaining a reliable non-outlier subset. Simulation studies and real data analysis show that our new method can provide more reliable results than the existing meta-analysis methods in the presence of outliers.

## 1. Introduction

With the advances in biological sciences, omics data have been playing an important role in many different fields of research. A typical example of such data includes gene expression data targeting for the identification of important genes that are related to disease status or clinical outcomes (Zhao et al., 2015). Nevertheless, as biological experiments are often measured with a relatively small number of samples, many identified genes are in fact very sensitive to mild data perturbations and thus lack of reliability. From another perspective, since many publicly available datasets have addressed the same scientific problems, one may consider to integrate multiple sources of data to borrow information across the studies and so improve the model interpretation and boost the statistical power (Glass, 1976; Wu et al., 2019). As an example, the integration analysis of genomic data from multiple studies has discovered new loci that are related to diseases including childhood obesity, colorectal cancer, and Crohn's disease (Houlston et al., 2008).

Meta-analysis is an efficient tool for integrating the scientific results from multiple studies. The classical meta-analysis methods are mainly based on the summary statistics including the *p*-values (Li and Tseng, 2011; Zhang et al., 2020) and/or the effect sizes (Choi et al., 2003; Chang et al., 2013). Recently, He et al. (2016) proposed a sparse method for meta-analyzing high-dimensional regression coefficients, which is based solely on the estimates of coefficients from multiple studies. When raw data from multiple studies are available, as recommended by Tang and Song (2016), a retreat to the classical meta-analysis methods is often necessary. Specifically, under such circumstances, it becomes possible to jointly assess the effect of selected covariates at the study and group levels, which can incorporate heterogeneous effects from different studies so as to outperform the classical meta-analysis with better estimation accuracy (George, 2019).

Due to the high-dimensionality of omics data, the number of genes is often larger than the sample size. Incorporation of variable selection into raw data analysis has been one hot topic in statistics. For example, Zhou and Zhu (2010) proposed a bi-level variable selection method for selecting important genes, which not only removes unimportant groups efficiently but also maintains the flexibility of selecting variables within the group. When the heterogeneity exists between multiple studies, however, the important genes may only be remarkable in some studies but not in others. In view of this, Li et al. (2014) further extended the bi-level variable selection to heterogeneous high-dimensional multiple datasets. They treated the coefficients of each covariate from all datasets as groups, and performed the simultaneously variable selection both on the group and within the group. For other existing variable selection methods including, for example, group Bridge, composite MCP, and group exponential lasso that can be extended to meta-analyzing multiple studies, one may refer to Zhao et al. (2015), Kim et al. (2017), and Rashid et al. (2020).

Despite the huge popularity of variable selection methods in meta-analysis, little attention has been paid to the extension of these methods to handle outliers in high-dimensional data (Chi and Scott, 2014). For biological data, it is not uncommon that the tissue samples are mislabeled or contaminated (Wu et al., 2019). Outliers may strongly influence the accuracy of parameter estimation and variable selection, and as shown in Alfons et al. (2013), even one single outlier has the potential to make the selected variables based on the lasso penalty completely unreliable. This motivates us to consider the robust alternatives, especially when integrating the multiple datasets collected from different platforms and laboratories. Needless to say, robust estimation has a long history under the classical paradigm where the sample size is large and the dimension is small, see, for example, Yohai (1987), Hadi and Simonoff (1993), and Bianco and Yohai (1996). In particular, Rousseeuw and Leroy (1987) proposed a least trimmed squares estimator (LTS), which was shown to have a high breakdown point and was further improved by the well-designed fast algorithm (Fast-LTS) in Rousseeuw and Driessen (2006). More recently, Alfons et al. (2013) and Yang et al. (2018) extended LTS to high-dimensional data with the alternating minimization algorithm. Ren et al. (2019) investigated a robust variable selection for continuous censored data, where the least absolute deviation loss was adopted to accommodate heavy-tailed data. For a review of recent developments on robust regression and variable selection methods, one may refer to Wu and Ma (2015) and Sun et al. (2020).

We note, however, that the aforementioned robust methods have all been focused on a single study. Moreover, most of the existing methods are based on robust loss functions that aim to deal with heavy-tailed continuous data; see, for example, the least absolute deviation and check loss functions (Wu and Ma, 2015). In recent public biological database (e.g., Gene Expression Omnibus database), many datasets are collected from case-control studies with binary phenotypes. Therefore, the commonly used robust loss functions may not be directly applicable to this scenario. In this paper, inspired by the idea of the LTS estimator and the bi-level lasso variable selection (Zhou and Zhu, 2010; Li et al., 2014), we propose a two-step procedure for the robust variable selection that can be applied to meta-analyzing multiple case–control studies. In the first step, we search a clean index subset for each study based on the Fast-LTS algorithm and the bi-level variable selection technique. In the second step, we further refine a reweighting rule to enhance the estimation efficiency and the accuracy of variable selection. The key idea in this step is to identify outliers according to the current model obtained in the first step and to assign a small or zero weight for outliers. Our new robust meta-analysis method has two main advantages: (1) the Fast-LTS algorithm guarantees the convergence of the selected clean subsets; (2) the bi-level variable selection not only identifies important covariates with the strength of multiple datasets, but also maintains the flexibility of variable selection between the datasets to account for the data heterogeneity. Consequently, in the presence of outliers, our proposed method can provide better parameter estimation and also identify more accurate informative covariates than the existing strategies, especially when the dimension is large.

The rest of this paper is organized as follows. In section 2, we describe the model setting and develop the new algorithm for our two-step robust meta-analysis method. The selection of tuning parameters involved in the algorithm is also discussed. In section 3, we conduct simulation studies to assess the performance of the our robust estimation in meta-analyzing multiple datasets. We further apply the new method to robustly analyze a real data example in section 4. Finally, we conclude the paper with some future work in section 5, and provide the technical results in the Appendix.

## 2. Methods and Algorithm

In this section, we first formulate the model in section 2.1, then propose a two-step robust meta-analysis method in section 2.2, and finally, we present the selection of tuning parameters in section 2.3.

### 2.1. Data and Models

Suppose there are *M* independent studies, and each study contains *n*_*k*_ subjects for *k* = 1, …, *M*. Let also $D_{k}=\left\{\left(x_{ki},y_{ki}\right),i=1,\ldots ,n_{k}\right\}$ be the raw data, where *y*_*ki*_ ∈ {0, 1} is a binary response variable and $x_{ki}={\left(x_{ki,1},\ldots ,x_{ki,p}\right)}^{T}\in R^{p}$ is the covariate vector. Throughout this paper, we assume that the dimension *p* is common for all the studies. To link *y*_*ki*_ to *****x*****_*ki*_, we consider the logistic model with

(2.1) $$\begin{array}{l} \pi_{ki}=P\left(y_{ki}=1|x_{ki}\right)=\frac{\exp\left(\beta_{k0}+x_{ki}^{T}\beta_{k}\right)}{1+\exp\left(\beta_{k0}+x_{ki}^{T}\beta_{k}\right)}, \end{array}$$

where $\beta_{k}={\left(\beta_{k1},\ldots ,\beta_{kp}\right)}^{T}\in R^{p}$ is the unknown coefficient vector for the *k*th study that captures the effect of each covariate. Since the intercept β_*k*0_ can be readily handled, without loss of generality, we will suppress it for convenience. To model the heterogeneity between the studies, we allow **β**_*k*_ to vary with *k*. For omics data, as mentioned earlier, the number of covariates *p* is often much larger than the sample size *n*, and meanwhile only a small proportion of covariates will be related to the response variable. We divide the covariates into two disjoint sets: the informative set *I*_*k*1_ = {*j* = 1, …, *p*:β_*kj*_ ≠ 0} and the noninformative set *I*_*k*1_ = {*j* = 1, …, *p*:β_*kj*_ = 0} for *k* = 1, …, *M*. Our main goals are to identify the informative sets and to estimate the coefficients of the informative covariates.

Note that each covariate has *M* coefficients across the studies. When the *M* datasets come from studies that focus on the same biological questions, the *M* coefficients may share some common information. This makes it possible to integrate information across multiple datasets and make simultaneous coefficient estimation and variable selection. On the other side, however, outliers and data contamination have been widely observed in the predictors and responses, and as a consequence, they will yield the lasso-type penalties largely unreliable.

### 2.2. Robust Meta-Analysis Method

In this section, we propose a new two-step procedure for robustly meta-analyzing multiple omics data.

#### 2.2.1. Simultaneous Estimation

Let *H*_*k*_ ⊆ {1, 2, …, *n*_*k*_} be a subset of the indexes from the *k*th study with the cardinality |*H*_*k*_| = *h*_*k*_ for *k* = 1, …, *M*, and $H=\left\{H_{1},\ldots ,H_{M}\right\}$ be a subset of the indexes for the *M* studies. Then by following Zhou and Zhu (2010) and Li et al. (2014), we define the objective function as

(2.2) $$\begin{array}{l} Q\left(H,\beta \right)=\overset{M}{\underset{k=1}{\sum }}\underset{i\in H_{k}}{\sum }d\left(x_{ki}^{T}\beta_{k},y_{ki}\right)+\lambda \overset{p}{\underset{j=1}{\sum }}{\left(\overset{M}{\underset{k=1}{\sum }}|\beta_{kj}|\right)}^{1/2}, \end{array}$$

where $\beta ={\left(\beta_{1}^{T},\ldots ,\beta_{M}^{T}\right)}^{T}$ is the stack of the coefficient vectors, and

(2.3) $$\begin{array}{l} d\left(x_{ki}^{T}\beta ,y_{ki}\right)=-y_{ki}\log\pi_{ki}-\left(1-y_{ki}\right)\log\left(1-\pi_{ki}\right) \end{array}$$

is the deviance. When the set H is outlier-free, minimizing the objective function (2.2) gives the robust and sparse estimator for the coefficients as

$${\hat{\beta }}_{H}={\left({\hat{\beta }}_{1}^{T},\ldots ,{\hat{\beta }}_{M}^{T}\right)}^{T}={\arg\min}_{\beta }Q\left(H,\beta \right),$$

where ${\hat{\beta }}_{k}={\left({\hat{\beta }}_{11},\ldots ,{\hat{\beta }}_{1p}\right)}^{T}$ is the estimate of the coefficient vector in the *k*th study.

Note that the square root penalty (or *L*_1/2_ norm) in (2.2) treats β_1*j*_, …, β_*Mj*_ as a group for each covariate *j*, and conducts a group-type variable selection. In addition, the *L*_1_ norm used inside the square root penalty can perform a study-specific variable selection that shrinks the small coefficients to zero and keeps only the large coefficients (Tsybakov and Vande, 2005). Then, in total, the penalty term in (2.2) essentially plays a role for the bi-level variable selection; that is, it cannot only borrow common information across the studies, but also take into account the data heterogeneity and maintain the flexibility of parameter estimation between the studies. From this perspective, with the penalty term in (2.2), the optimization procedure actually borrows the strength across the *M* studies and is quite different from performing a separate variable selection in each individual study (Li et al., 2014).

In practice, to determine a set that can well approximate the outlier-free set H, it will involve iteratively optimizing the objective function (2.2). Note also that the square root penalty in (2.2) is not a convex function and has a complex nonlinear form. To solve the problem, we first give a simpler and equivalent version for the optimization.

**THEOREM 1**. *Let β_*kj*_ = α_*j*_ γ _*kj*_ for *k* = 1, …, *M* and *j* = 1, …, *p*. Let also* $\alpha ={\left(\alpha_{1},\ldots ,\alpha_{p}\right)}^{T}$ and $\gamma_{k}={\left(\gamma_{k1},\ldots ,\gamma_{kp}\right)}^{T}$. *Consider the following objective function:*

(2.4) $$\begin{array}{l} Q_{1}\left(H,\alpha ,\gamma \right)=\overset{M}{\underset{k=1}{\sum }}\underset{i\in H_{k}}{\sum }d\left(x_{ki}\beta_{k},y_{ki}\right)+\overset{p}{\underset{j=1}{\sum }}|\alpha_{j}|+\lambda_{1}\overset{M}{\underset{k=1}{\sum }}\overset{p}{\underset{j=1}{\sum }}|\gamma_{kj}|, \end{array}$$

*where* H *is a set of indexes as in (2.2) and* $\gamma ={\left(\gamma_{1}^{T},\ldots ,\gamma_{M}^{T}\right)}^{T}$. *For the minimization problems of (2.2) and (2.4) with tuning parameter* $\lambda_{1}=\lambda^{2}/4$, (*a*) *if* $\left({\hat{\alpha }}_{H},{\hat{\gamma }}_{H}\right)$ *is a solution of (2.4), then* ${\hat{\beta }}_{H}$ *with* ${\hat{\beta }}_{kj}={\hat{\alpha }}_{j}{\hat{\gamma }}_{kj}$ *is a solution of (2.2); and* (*b*) *if* ${\hat{\beta }}_{H}$ *is a solution of (2.2), then there exists a solution* $\left({\hat{\alpha }}_{H},{\hat{\gamma }}_{H}\right)$ *of (2.4) such that* ${\hat{\beta }}_{kj}={\hat{\alpha }}_{j}{\hat{\gamma }}_{kj}$.

The proof of Theorem 1 is given in Appendix A. This theorem further verifies that the penalty term in (2.2) performs a bi-level variable selection. By a decomposition of β_*kj*_, the parameter α_*j*_ controls the sparsity of the *j*th group β_1*j*_, …, β_*Mj*_, and γ_*kj*_ reflects the sparsity within the *j*th group. If α_*j*_ is shrunk to zero, all β_*kj*_, *k* = 1, …, *M* in the *j*th group will be zero. Since the objective function (2.4) only has two lasso penalties without interaction, Zhou and Zhu (2010) and Li et al. (2014) applied the lasso algorithm to solve **α** and **γ**, iteratively. Moreover, they have also implemented this algorithm by the “glmnet” in the R software.

Next, to find an approximate outlier-free subset for the *M* studies, we propose to combine the bi-level variable selection technique with Fast-LTS (Rousseeuw and Driessen, 2006; Alfons et al., 2013). We first introduce a definition that will be useful for the searching algorithm.

**DEFINITION 1**. *Let* ${\hat{\beta }}_{H}={\left({\hat{\beta }}_{1,H}^{T},\ldots ,{\hat{\beta }}_{M,H}^{T}\right)}^{T}$ *be the estimate of **β** based on the set* $H=\left\{H_{1},\ldots ,H_{M}\right\}$. *Then, an approximate clean subset for the *k*th study based on* H *is given as*

(2.5) $$\begin{array}{l} {\tilde{H}}_{k}|H={\arg\min}_{G\in {\tilde{G}}_{k}}\underset{i\in G}{\sum }d\left(x_{ki}^{T}{\hat{\beta }}_{k,H},y_{ki}\right), \end{array}$$

*where* ${\tilde{G}}_{k}=\left\{G:G\subseteq \left\{1,\ldots ,n_{k}\right\}\text{and }|G|=h_{k}\right\}.$ *Furthermore, an approximate clean subset for the *M* studies based on* H *is defined as* $\tilde{H}|H=\left\{{\tilde{H}}_{1},\ldots ,{\tilde{H}}_{M}\right\}$.

Accordingly, let $H_{0}$ and ${\hat{\beta }}_{H_{0}}$ be the initial subset for the studies and the corresponding estimate of **β**, respectively. By using (2.5) recursively, we can obtain the approximate clean subset for the *k*th study in the *t*th iteration, denoted as *H*_*k,t*_. Consequently, the approximate clean subset for all studies in the *t*th iteration is given as $H_{t}=\left\{H_{1,t},\ldots ,H_{M,t}\right\}$. A similar procedure was also adopted in Rousseeuw and Driessen (2006) and Alfons et al. (2013); that is, selecting a subset with minimal deviance may gradually exclude outlier samples.

**THEOREM 2**. *For any given initial set* $H_{0}$, *by recursively applying (2.5), we have*

$$Q\left(H_{t+1},{\hat{\beta }}_{H_{t+1}}\right)\leq Q\left(H_{t},{\hat{\beta }}_{H_{t}}\right).$$

This theorem, with the proof in Appendix A, shows that the objective function decreases in each iteration. Since there are only a finite number of index subsets of the collected observations, we can obtain a decreasing finite-length sequence, e.g., *Q*_1_ ≥ *Q*_2_ ≥ ⋯ ≥ *Q*_*t*_*M*__ with $Q_{t}=Q\left(H_{t},{\hat{\beta }}_{H_{t}}\right)$, this shows that a convergence can be reached after a finite number of iterations (Rousseeuw and Driessen, 2006; Alfons et al., 2013). For convenience, we refer to the searching procedure in (2.5) as the concentration step (*C*-step). Note that the selected subset after convergence of the *C*-step is related to the initial subset; to alleviate this problem, we perform this searching procedure with several different initial subsets as in Alfons et al. (2013). Throughout this paper, we consider 500 initial sets as $H_{0}^{\left(s\right)}=\left\{H_{1,0}^{\left(s\right)},\ldots ,H_{M,0}^{\left(s\right)}\right\}$ for *s* = 1, …, 500, where $H_{k,0}^{\left(s\right)}$ is the initial subset for the *k*th study. To construct $H_{k,0}^{\left(s\right)}$, we adopt a similar procedure as in Kurnaz et al. (2018), where the indexes of four observations from the *k*th study are randomly selected, two from each of the groups. This construction method leads to a high possibility of having no outliers in the initial subsets.

Assume that $H_{s}^{*}=\left\{H_{s,1}^{*},\ldots ,H_{s,M}^{*}\right\}$ is the converged approximate clean subset based on $H_{0}^{\left(s\right)}$ and ${\hat{\beta }}_{H_{s}^{*}}={\left({\hat{\beta }}_{1,H_{s}^{*}},\ldots ,{\hat{\beta }}_{M,H_{s}^{*}}\right)}^{T}$ is the resulting coefficient estimate. Then for the *k*th study, the index of the best clean subset among $H_{1,k}^{*},\ldots ,H_{500,k}^{*}$ can be given as

$$s_{k}^{*}=\arg\underset{s\in \left\{1,\ldots ,500\right\}}{\min}\underset{i\in H_{s,k}^{*}}{\sum }\psi \left(x_{ki}^{T}{\hat{\beta }}_{k,H_{s}^{*}},y_{ki}\right)\;for\;k=1,\ldots ,M,$$

where ψ(*x, y* = 0) = ϕ(*x*), ψ(*x, y* = 1) = ϕ(−*x*), and ϕ(*x*) is given in Definition A1 of the Appendix. As mentioned in Bianco and Yohai (1996) and Crous and Haesbroeck (2003), the function ψ(*x, y*) provides a robust loss measure for binary classification, which assigns a nearly zero score to the points with correct classification and a high score to the points with misclassification. Hence, the best clean subset for the *k*th study indicates the lowest classification loss among all those identified clean subsets for this study. Finally, the best clean set for the *M* studies is given as $H^{*}=\left\{H_{s_{1}^{*},1}^{*},\ldots ,H_{s_{M}^{*},M}^{*}\right\}.$

Also, in view of the heavy computation in the *C*-step on each of the 500 initial subsets. As alternative, we propose an alternative to perform two *C*-steps and find the best 10 subsets for the *M* studies as initial subsets. The rest searching procedure is similar as above paradigm. To summarize, we have the new algorithm as follows.

1. Let $H_{o}^{\left(s\right)}=\left\{H_{1,o}^{\left(s\right)},\ldots ,H_{M,o}^{\left(s\right)}\right\}$ be the initial sets for *s* = 1, 2, …, 500.
2. Let $H=H_{o}^{\left(s\right)}$ and compute the estimator for **β** by minimizing (2.4), denoted as ${\hat{\beta }}_{H_{o}^{\left(s\right)}}={\left({\hat{\beta }}_{1,H_{o}^{\left(s\right)}}^{T},\ldots ,{\hat{\beta }}_{M,H_{o}^{\left(s\right)}}^{T}\right)}^{T}$.
3. Search the approximate clean subset for the *k*th study as
  $$H_{k,1}^{\left(s\right)}={\arg\min}_{G\in {\tilde{G}}_{k}}\underset{i\in G}{\sum }d\left(x_{ki}^{T}{\hat{\beta }}_{k,H_{o}^{\left(s\right)}},y_{ki}\right),$$
  where ${\tilde{G}}_{k}$ is the index set as in (2.5). The approximate clean subset for the *M* studies is $H_{1}^{\left(s\right)}=\left\{H_{1,1}^{\left(s\right)},\ldots ,H_{M,1}^{\left(s\right)}\right\}$.
4. Repeat Step 2 on $H=H_{1}^{\left(s\right)}$. Let also ${\hat{\beta }}_{H_{1}^{\left(s\right)}}={\left({\hat{\beta }}_{1,H_{1}^{\left(s\right)}}^{T},\ldots ,{\hat{\beta }}_{M,H_{1}^{\left(s\right)}}^{T}\right)}^{T}$ be the corresponding coefficient estimate.
5. For $H_{k,1}^{\left(s\right)}\in H_{1}^{\left(s\right)}$ with *s* = 1, …, 500 and *k* = 1, …, *M*, let
  $$e_{ks}=\underset{i\in H_{k,1}^{\left(s\right)}}{\sum }\psi \left(x_{ki}^{T}{\hat{\beta }}_{k,H_{1}^{\left(s\right)}},y_{ki}\right).$$
  Search a subset of indexes such that {π_*k*, 1_, …, π_*k*, 10_} ⊂ {1, …, 500} with *e*_*k*,_π__*k*, 1__ ≤ … ≤ *e*_*k*,_π__*k*, 10__. The best 10 sets among $H_{1}^{\left(1\right)},\ldots ,H_{1}^{\left(500\right)}$ are given as ${\tilde{H}}_{1}^{\left(s\right)}=\left\{H_{1,1}^{\left(\pi_{1,s}\right)},\ldots ,H_{M,1}^{\left(\pi_{M,s}\right)}\right\}$ for *s* = 1, …, 10.
6. Let $H={\tilde{H}}_{1}^{\left(s\right)}$ be the initial set for *s* = 1, …, 10, respectively, and repeat Steps 2–3 for a total of *t* times until convergence such that $||{\hat{\beta }}_{H_{t}^{\left(s\right)}}-{\hat{\beta }}_{H_{t-1}^{\left(s\right)}}|{|}_{2}\leq \epsilon$, where ||·||_2_ is the Euclidean norm and ϵ is a pre-specified small constant. The converged approximate clean subset and the coefficient estimate for all *M* studies are denoted as $H_{s}^{*}=\left\{H_{s,1}^{*},\ldots ,H_{s,M}^{*}\right\}$ and ${\hat{\beta }}_{H_{s}^{*}}={\left({\hat{\beta }}_{1,H_{s}^{*}}^{T},\ldots ,{\hat{\beta }}_{M,H_{s}^{*}}^{T}\right)}^{T}$, respectively.
7. For $H_{s,k}^{*}\in H_{s}^{*}$ with *s* = 1, …, 10 and *k* = 1, …, *M*, let
  $$s_{k}^{*}=\arg\underset{s\in \left\{1,\ldots ,10\right\}}{\min}\underset{i\in H_{s,k}^{*}}{\sum }\psi \left(x_{ki}^{T}{\hat{\beta }}_{k,H_{s}^{*}},y_{ki}\right).$$
  The best clean subset for all *M* studies is given as $H^{*}=\left\{H_{s_{1}^{*},1}^{*},\ldots ,H_{s_{M}^{*},M}^{*}\right\},$ and the corresponding estimate of **β** is ${\hat{\beta }}_{H^{*}}={\left({\hat{\beta }}_{1,H^{*}}^{T},\ldots ,{\hat{\beta }}_{M,H^{*}}^{T}\right)}^{T}$.

Finally, we observe that in the first several *C*-steps, the algorithm for minimizing (2.4) may not stable. For this, we may restrict that α_1_ = ⋯ = α_*p*_ = 1.

#### 2.2.2. Reweighting Step

Note that the LTS-type estimator only uses a subset of data and may suffer from a low efficiency. To further improve the model estimation, Kurnaz et al. (2018) proposed a reweighting step such that the identified outliers based on the current estimated model will be assigned with a small or zero weight. For our robust meta-analysis method, we adopt a similar reweighting procedure, which is based on the Pearson residuals ${\hat{r}}_{ki}=\left(y_{ki}-{\hat{\pi }}_{ki}\right)/\sqrt{{\hat{\pi }}_{ki}\left(1-{\hat{\pi }}_{ki}\right)}$, where ${\hat{\pi }}_{ki}=\exp\left(x_{ki}^{T}{\hat{\beta }}_{k,H^{*}}\right)/\left[1+\exp\left(x_{ki}^{T}{\hat{\beta }}_{k,H^{*}}\right)\right]$ is the conditional probability of the logistic model. Since *r*_*ki*_ is a standardized statistic and is approximately normally distributed, the weights for the observations are given as

(2.6) $$\begin{array}{l} {\hat{w}}_{ki}=\{\begin{array}{ll} 0, & \left|{\hat{r}}_{ki}\right|>\Phi^{-1}(1-\delta ),\\ 1, & \left|{\hat{r}}_{ki}\right|\leq \Phi^{-1}(1-\delta ), \end{array}\;\text{for}\;\;k=1,\ldots ,M\;\;\text{and}\;\\ \;i=1,\ldots ,n_{k},n \end{array}$$

where Φ is the cumulative distribution function of the standard normal distribution. Throughout this paper, we select δ = 0.0125 as in Alfons et al. (2013) and Kurnaz et al. (2018) such that 2.5% of the observations from the standard normal distribution are considered as outliers. The reweighed objective function is

(2.7) $$\begin{array}{l} Q_{w}\left(\beta \right)=\overset{M}{\underset{k=1}{\sum }}\overset{n_{k}}{\underset{i=1}{\sum }}{\hat{w}}_{ki}d\left(x_{ki}^{T}\beta_{k},y_{ki}\right)+\lambda \overset{p}{\underset{j=1}{\sum }}{\left(\overset{M}{\underset{k=1}{\sum }}|\beta_{kj}|\right)}^{1/2}, \end{array}$$

Consequently, the robust estimator for meta-analyzing multiple studies is given as

$$\tilde{\beta }={\left({\tilde{\beta }}_{1}^{T},\ldots ,{\tilde{\beta }}_{M}^{T}\right)}^{T}=\arg\underset{\beta }{\min}Q_{w}\left(\beta \right),$$

where ${\tilde{\beta }}_{k}$ is the estimate of the coefficient vector for the *k*th study. Obviously, when the data do not have outliers or only have a small proportion of outliers, the reweighing procedure uses more observations and hence may improve the estimation accuracy.

**Figure 1:**
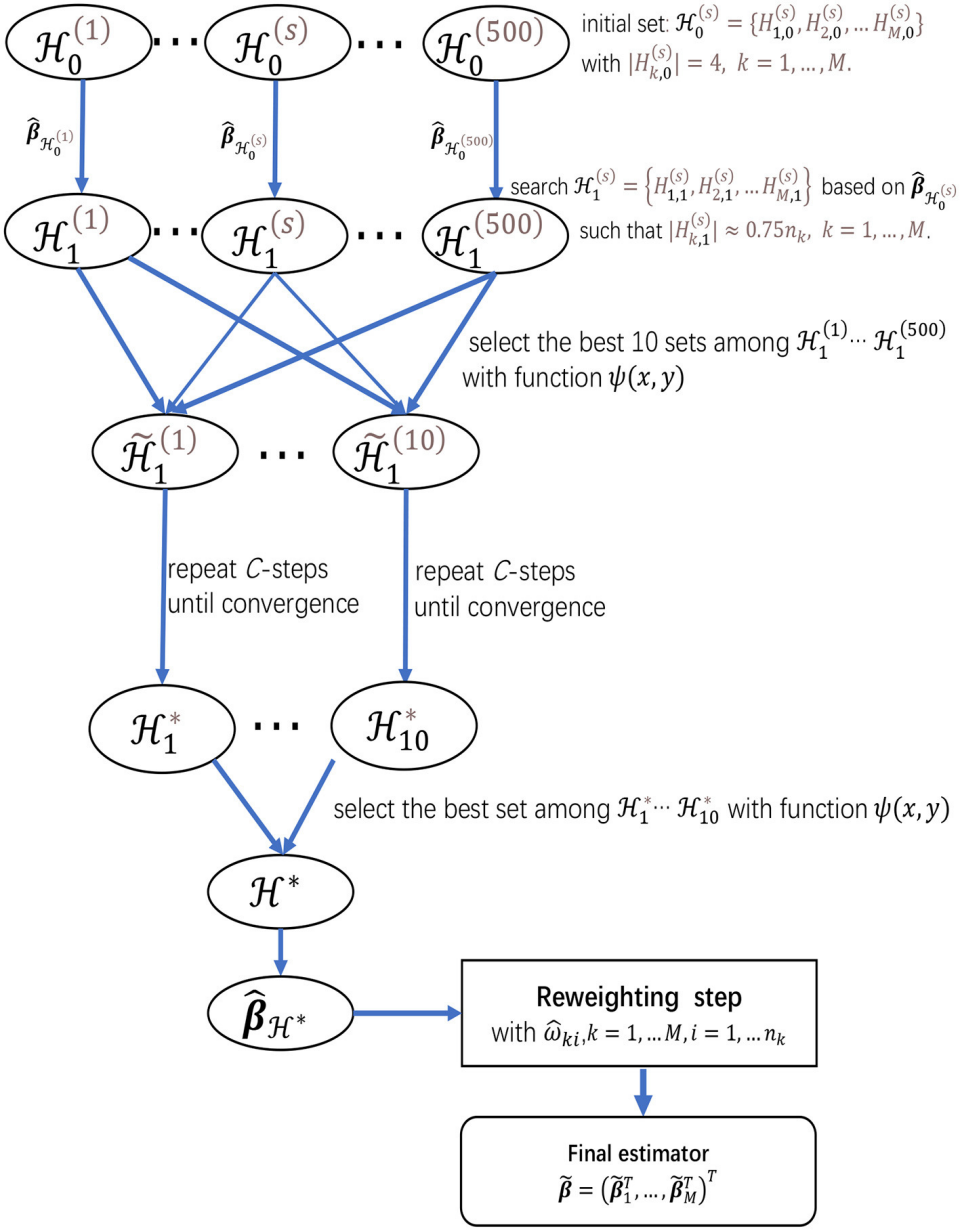
The flow chart of the two-step procedure for meta-analyzing multiple studies, which provides a summarization for the searching procedure for $H^{*}$ and the reweighting step.

Finally, to give more insights on the algorithms in sections 2.2.1 and 2.2.2, we present a flow chart of the two-step procedure for meta-analyzing multiple studies in Figure 1, which provides a summary for the searching procedure for $H^{*}$ and the reweighting step.

### 2.3. Selection of the Tuning Parameters

In section 2.2, we need to pre-specify the cardinalities *h*_1_, …, *h*_*M*_ before searching the approximate clean subset ${\tilde{H}}^{*}$. If some studies contain a large proportion of outliers, then the cardinalities of the selected subsets from the studies ought to be small, e.g., *h*_*k*_ ≈ *n*_*k*_/2, and vice versa. In practice, if we do not have prior knowledge for the number of outliers, we recommend to use *h*_*k*_ ≈ 0.75*n*_*k*_ as adopted in Rousseeuw and Driessen (2006), Alfons et al. (2013), and Kurnaz et al. (2018).

Note that the optimization problems in (2.2) and (2.7) can be rewritten as (2.4), and hence we only need to select the tuning parameter in (2.4). Various data-driven techniques have been well developed in the literature including, for example, the cross-validation, the generalized cross-validation, and the Bayesian information criterion (BIC) procedures. We adopt the BIC to select the tuning parameter as recommended in Alfons et al. (2013). Specifically, we compute the BIC after obtaining $H^{*}$ in the *C*-steps, which is given as

(2.8) $$\begin{array}{l} \text{BIC}\left(\lambda_{1}\right)=\overset{M}{\underset{k=1}{\sum }}\left\{-2L_{k}\left({\hat{\beta }}_{k,H^{*}},H^{*}\right)+\text{df}\left({\hat{\beta }}_{k,H^{*}}\right)\log\left(h_{k}\right)\right\}, \end{array}$$

where $L_{k}\left({\hat{\beta }}_{k,H^{*}},H^{*}\right)=\overset{H_{k}}{\underset{i=1}{\sum }}d\left(x_{ki}{\hat{\beta }}_{k,H^{*}},y_{ki}\right)$ with $H_{k}\in H^{*}$, and $\text{df}\left({\hat{\beta }}_{k,H^{*}}\right)$ is the number of non-zero components in ${\hat{\beta }}_{k,H^{*}}$. A similar procedure is also performed in the reweighting procedure to select the tuning parameter.

## 3. Numerical Studies

In this section, we conduct simulations to evaluate the numerical performance of our new robust lasso-type meta-analysis method (RL-meta) and compare it with some existing methods. Specifically, we consider the state-of-the-art methods from Li et al. (2014), Alfons et al. (2013), and Friedman et al. (2010). Noting that the latter two methods perform the variable selection on each study individually, hence for convenience, we refer to them as L-meta, RL-each, and L-each, respectively.

Let TP, FP, and FN be the number of true positives, false positives, and false negatives, respectively. Then to evaluate the performance of these methods, we consider four criteria including Precision = TP/(TP+FP), Recall = TP/(TP+FN), the *F*_1_ score (*F*_1_), and the root mean squared error (RMSE), where

$$\begin{array}{l} F_{1}=\frac{2\times \text{Presicion}\times \text{Recall}}{\text{Presicion}+\text{Recall}}\text{ and }\\ \text{RMSE}=(\frac{1}{M}\overset{M}{\underset{k=1}{\sum }}\overset{p}{\underset{j=1}{\sum }}{\left({\hat{\beta }}_{kj}-\beta_{kj}\right)}^{2}{)}^{1/2}. \end{array}$$

Note that Precision, Recall, and *F*_1_ all range from 0 to 1, which measure the accuracy of variable selection with a larger value being preferred. As an addition, RMSE measures the loss of coefficient estimation, for which a small value is favorable.

### 3.1. Clean Data

In the first simulation, we consider clean data with no outliers. Specifically, we generate *M* studies and each has *n* observations. The covariate vector $x_{ki}={\left(x_{ki1},\ldots ,x_{kip}\right)}^{T}$ are randomly sampled from the multivariate normal distribution *N*(**0**, *****I*****_*p*_) for *k* = 1, …, *M* and *i* = 1, …, *n*, where *****I*****_*p*_ is the identity matrix. Then the response variables are generated as *y*_*ki*_ = 1 if *****x*****_*ki*_**β**_*k*_ + ε_*ki*_ > 0, otherwise, *y*_*ki*_ = 0, where $\beta_{k}={\left(\beta_{k1},\ldots ,\beta_{kp}\right)}^{T}$, ε_*ki*_ is the independent noise sampled from *N*(0, 1). To access the performance of our RL-meta under different levels of heterogeneity, we let β_*kj*_ = *z*_*kj*_*b*_*kj*_ for *k* = 1, …, *M* and *j* = 1, …, 10 and β_*kj*_ = 0 for *j* = 11, …, *p*, where *z*_*kj*_ is randomly sampled from a Bernoulli distribution with the success rate π_0_ and *b*_*kj*_ is randomly sampled from *N*(1.5, 0.5^2^). This means that only the first 10 covariates may be possibly related to the response variable in each study, it is informative with probability π_0_ and uninformative with probability 1 − π_0_. We consider π_0_ = 0.2, 0.5, or 0.9 to represent three levels of heterogeneity between the studies. In addition, we also consider (*n* = 100, *p* = 50) or (*n* = 150, *p* = 1, 000) as a low or large dimension, respectively, and the numbers of studies as *M* = 2 or 8.

**Figure 2:**
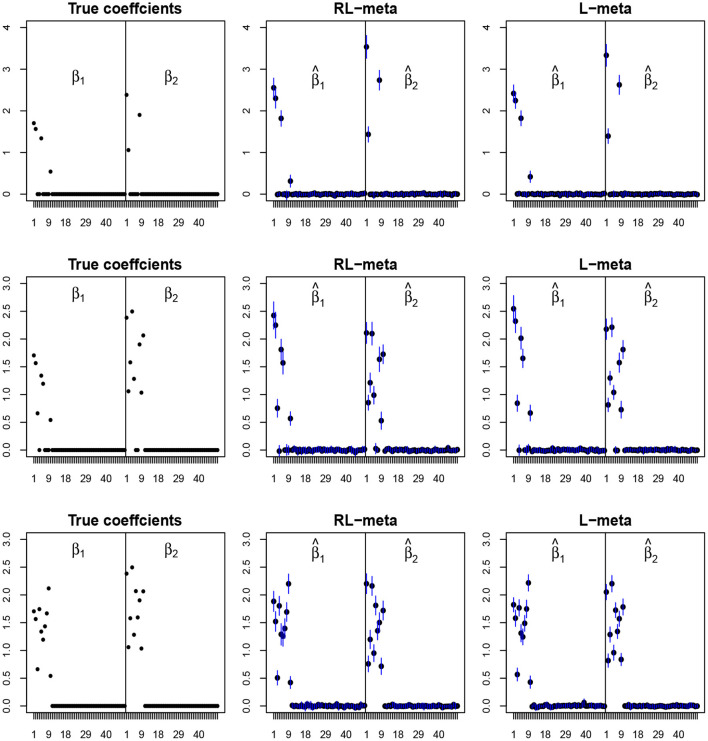
The coefficient estimates with clean data for *M* = 2 and (*n, p*) = (100, 50). The blue points and lines represent the mean values and the interval estimates of coefficients over 100 simulations. Rows from top to bottom correspond to π_0_ = 0.2, 0.5, 0.9, respectively.

**Figure 3:**
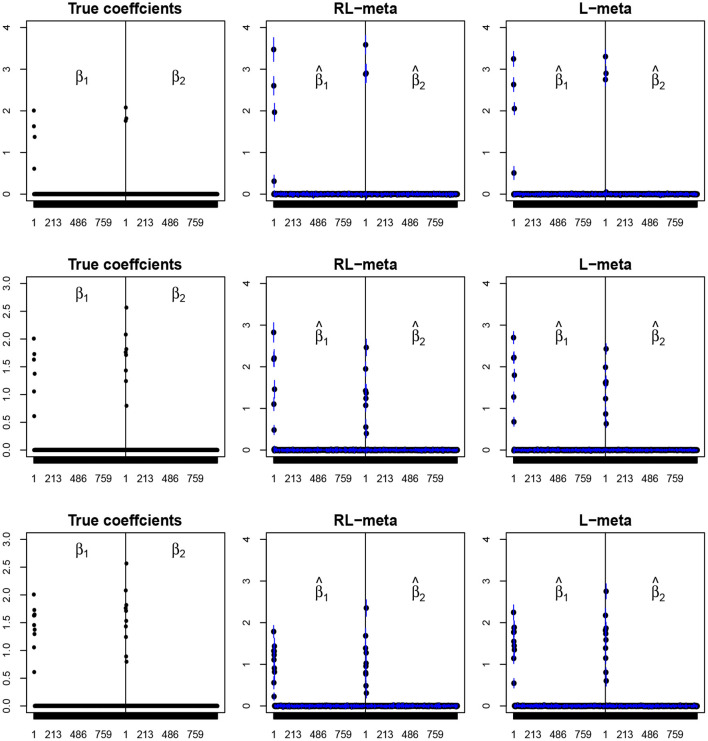
The coefficient estimates with clean data for *M* = 2 and (*n, p*) = (150, 1, 000). The blue points and lines represent the estimated values and the interval estimates of coefficients over 100 simulations. Rows from top to bottom correspond to π = 0.2, 0.5, 0.9, respectively.

To visualize the coefficient estimation for more insights, we plot the average values of the estimates for each coefficient with the confidence intervals (mean ±3× standard error) for *M* = 2 studies (see Figures 2, 3). To save space, we move the plots of L-each and RL-each to Appendix B (see Figures S1, S2). When there is no outlier, RL-meta and L-meta both provide good estimates for the coefficients, where they are close to the true coefficients especially with a low dimension (e.g., *p* = 50). Figure A1 shows that RL-each and L-each can provide an accurate variable selection, whereas the estimates for nonzero coefficients tend to be smaller than the true coefficients, especially when the dimension is larger than the sample size. This phenomenon was also observed in Zhao and Yu (2006), where they showed that the convex penalty often shrinks the estimates of the nonzero coefficients too heavily. In contrast, since our RL-meta and L-meta both use a nonconvex regularization, they are able to reduce the estimation biases.

Tables 1, 2 show the scores of the measure criteria for each scenario with clean data. Based on the evaluation criteria, L-meta exhibits superiority than the other three methods, which has a higher Precision, Recall, and *F*_1_ in most settings. RL-meta is nearly as good as L-meta and is much better than L-each and RL-each. For example, when the dimension and the number of informative covariates tend to large, L-each and RL-each both exhibit an inflated RMSE, whereas RL-meta and L-meat still keep a low RMSE. This verifies that borrowing information across the studies can improve the estimation accuracy, especially when the dimension is large and the sample size is small.

**Table 1:** Results for low-dimensional data with clean data.

	**(******n**, **p******) = (100, 50)**				
***M* = 2**		**RL-meta**	**L-meta**	**RL-each**	**L-each**
π = 0.2	Precision	0.689 (0.019)	**0.714** (0.018)	0.623 (0.020)	0.563 (0.013)
	Recall	0.910 (0.007)	**0.934** (0.007)	0.762 (0.016)	0.909 (0.008)
	*F*_1_	0.769 (0.012)	**0.795** (0.012)	0.653 (0.010)	0.684 (0.011)
	RMSE	0.303 (0.011)	0.272 (0.010)	0.224 (0.003)	**0.202** (0.005)
π = 0.5	Precision	0.861 (0.008)	**0.879** (0.007)	0.806 (0.015)	0.708 (0.012)
	Recall	0.950 (0.006)	**0.976** (0.040)	0.358 (0.018)	0.763 (0.018)
	*F*_1_	0.820 (0.008)	**0.835** (0.007)	0.566 (0.010)	0.710 (0.008)
	RMSE	0.295 (0.005)	**0.253** (0.004)	0.578 (0.010)	0.456 (0.008)
π = 0.9	Precision	0.861 (0.008)	**0.879** (0.007)	0.806 (0.015)	0.708 (0.012)
	Recall	0.959 (0.006)	**0.978** (0.040)	0.358 (0.018)	0.727 (0.016)
	*F*_1_	0.900 (0.005)	**0.920** (0.045)	0.457 (0.015)	0.069 (0.010)
	RMSE	0.302 (0.005)	**0.259** (0.034)	0.706 (0.010)	0.643 (0.013)
***M* = 8**		**RL-meta**	**L-meta**	**RL-each**	**L-each**
π = 0.2	Precision	0.721 (0.009)	**0.729** (0.010)	0.597 (0.008)	0.531 (0.007)
	Recall	0.949 (0.004)	**0.953** (0.004)	0.767 (0.011)	0.942 (0.003)
	*F*_1_	0.815 (0.005)	**0.821** (0.005)	0.665 (0.006)	0.676 (0.006)
	RMSE	0.138 (0.002)	**0.110** (0.001)	0.179 (0.002)	0.141 (0.001)
π = 0.5	Precision	**0.706** (0.007)	0.691 (0.006)	0.658 (0.007)	0.588 (0.006)
	Recall	0.987 (0.002)	**0.992** (0.001)	0.626 (0.011)	0.928 (0.004)
	*F*_1_	**0.820** (0.005)	0.812 (0.004)	0.634 (0.006)	0.718 (0.005)
	RMSE	0.189 (0.003)	**0.176** (0.004)	0.306 (0.005)	0.266 (0.003)
π = 0.9	Precision	0.889(0.005)	**0.905** (0.005)	0.754 (0.008)	0.671 (0.006)
	Recall	0.992 (0.001)	**0.995** (0.007)	0.469 (0.002)	0.774 (0.011)
	*F*_1_	0.936 (0.003)	**0.947** (0.003)	0.578 (0.007)	0.712 (0.005)
	RMSE	0.209 (0.002)	**0.187** (0.001)	0.634 (0.005)	0.565 (0.011)

*The presented values are the means of Precision, Recall, F_1_, and RMSE with standard errors in parentheses, respectively, averaged over 100 simulations. The bold values for Presicion, Recall, and F1 score are the highest values, and the bold value for RMSE is the lowest value*.

**Table 2:** Results for high-dimensional data with clean data.

	**(******n**, **p******) = (150, 1, 000)**				
***M* = 2**		**RL-meta**	**L-meta**	**RL-each**	**L-each**
π = 0.2	Precision	0.536 (0.015)	0.489 (0.014)	0.633 (0.024)	**0.807** (0.015)
	Recall	0.900 (0.007)	**0.934** (0.007)	0.740 (0.016)	0.878 (0.006)
	*F*_1_	0.658 (0.011)	0.632 (0.012)	0.642 (0.013)	**0.833** (0.010)
	RMSE^*^	0.276 (0.047)	0.239 (0.071)	**0.208** (0.054)	0.226 (0.014)
π = 0.5	Precision	**0.747** (0.012)	0.665 (0.011)	0.716 (0.021)	0.787 (0.011)
	Recall	0.965 (0.005)	**0.974** (0.010)	0.321 (0.012)	0.684 (0.013)
	*F*_1_	**0.835** (0.007)	0.785 (0.007)	0.417 (0.011)	0.721 (0.009)
	RMSE^*^	0.253 (0.040)	**0.157** (0.089)	0.447 (0.091)	0.452(0.102)
π = 0.9	Precision	0.722 (0.012)	0.759 (0.011)	0.762 (0.025)	**0.790** (0.013)
	Recall	0.850 (0.010)	**0.975** (0.004)	0.178 (0.008)	0.448 (0.015)
	*F*_1_	0.778 (0.010)	**0.849** (0.007)	0.274 (0.010)	0.548 (0.012)
	RMSE^*^	0.261 (0.074)	**0.196** (0.014)	0.489 (0.075)	0.446 (0.071)
***M* = 8**		**RL-meta**	**L-meta**	**RL-each**	**L-each**
π = 0.2	Precision	0.694 (0.015)	0.709 (0.012)	0.573 (0.014)	**0.783** (0.008)
	Recall	**0.957** (0.004)	0.840(0.002)	0.630 (0.008)	0.863 (0.004)
	*F*_1_	0.796 (0.007)	0.811 (0.008)	0.587 (0.007)	**0.818** (0.004)
	RMSE^*^	0.321 (0.068)	**0.311** (0.075)	0.509 (0.063)	0.451 (0.045)
π = 0.5	Precision	0.687 (0.005)	**0.688** (0.005)	0.664 (0.014)	0.769 (0.007)
	Recall	0.988 (0.002)	**0.994** (0.001)	0.395 (0.008)	0.784 (0.006)
	*F*_1_	0.809 (0.004)	**0.812** (0.003)	0.483 (0.006)	0.774 (0.005)
	RMSE^*^	0.416 (0.064)	**0.316** (0.087)	0.447 (0.076)	0.435 (0.081)
π = 0.9	Precision	0.942 (0.002)	**0.952** (0.001)	0.712 (0.012)	0.760 (0.006)
	Recall	**0.988** (0.001)	0.964 (0.003)	0.198 (0.004)	0.473 (0.008)
	*F*_1_	**0.965** (0.002)	0.958 (0.002)	0.305 (0.004)	0.579 (0.007)
	RMSE^*^	0.574 (0.072)	**0.475** (0.031)	0.639 (0.064)	0.622 (0.105)

The presented values are the means of Precision, Recall, F_1_, and RMSE^*^ with standard errors in parentheses, respectively, averaged over 100 simulations. RMSE^*^= 10 × RMSE. The bold values for Presicion, Recall, and F1 score are the highest values, and the bold value for RMSE is the lowest value.

### 3.2. Contamination Data

In the second simulation, we consider contamination data with outliers. We randomly select *m*_0_ observations from each study and add outliers to those informative covariates. More specifically, for the observations with *y*_*ki*_ = 0 (or *y*_*ki*_ = 1), the informative covariates are replaced by values randomly sampled from *N*(5, 1). To avoid high-leverage points, those observations are assigned an opposite class label. That is, *y*_*ki*_ = 1 − δ(*****x*****_*ki*_**β**_*k*_ > 0), where δ(·) is an indicator function. Throughout this section, we fix *m*_0_ = 10, and the other parameter are the same as those in section 3.1.

**Figure 4:**
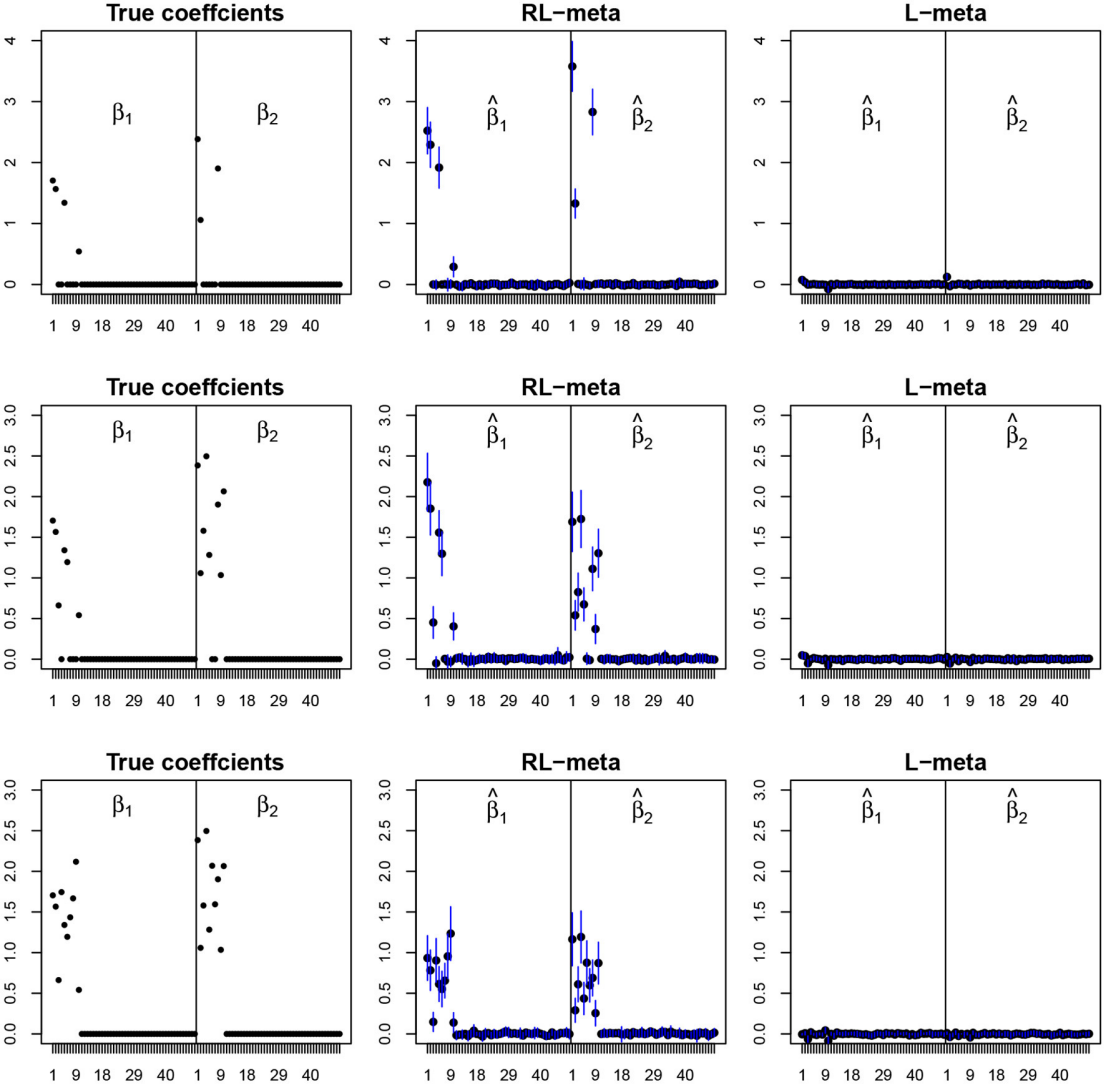
The coefficient estimates with contamination data for *M* = 2 and (*n, p*) = (100, 50). The blue points and lines represent the mean values and the interval estimates of coefficients over 100 simulations. Rows from top to bottom correspond to π_0_ = 0.2, 0.5, 0.9, respectively.

**Figure 5:**
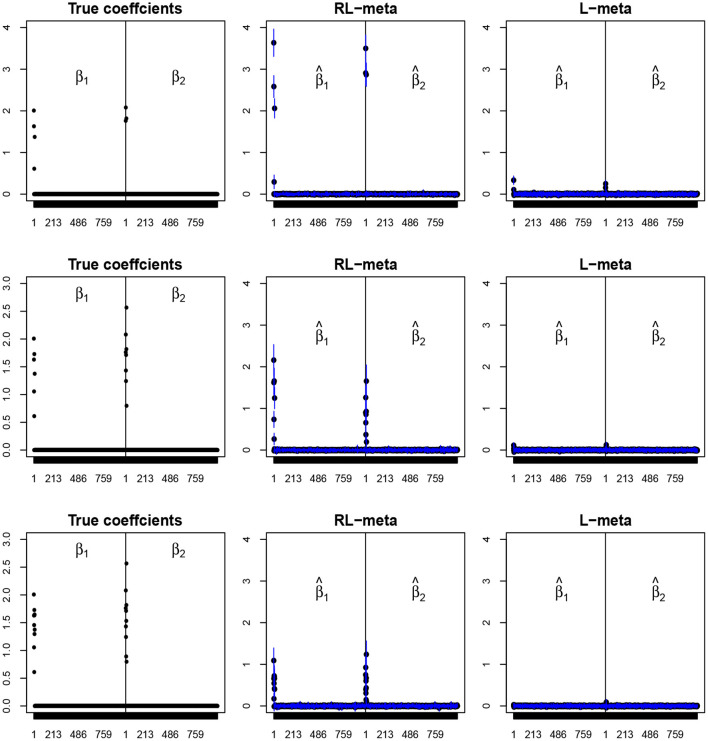
The coefficient estimates with contamination data for *M* = 2 and (*n, p*) = (150, 1, 000). The blue points and lines represent the estimated values and the interval estimates of coefficients over 100 simulations. Rows from top to bottom correspond to π = 0.2, 0.5, 0.9, respectively.

Figures 4, 5 plot the mean values of the estimates for each coefficient with the confidence intervals (mean ±3× standard error) for *M* = 2 studies under contamination data. To save space, we also move the plots of L-each and RL-each to Appendix B (see Figures S3, S4). From those figures, it is evident that RL-meta outperforms the other three methods in the presence of outliers. In particular, RL-meta and L-meta are able to select more informative covariates, whereas, L-meta and L-each both miss most informative variables, especially when the dimension is large. As we mentioned in the Introduction, this may due to the fact that classical lasso-type variable selection is sensitive to outliers and has a high-break down point.

Tables 3, 4 show the scores of the four measure criteria for each scenario under contamination data. RL-meta and RL-each both exhibit a higher Precision and Recall and a smaller RMSE than L-meta and L-each, especially when the number of informative covariates is large. This indicates that the two robust methods are able to identify more informative covariates and also keep a low false discovery rate when presenting outliers. When the number of studies and the number of informative variable are both small (e.g., *M* = 2 and π = 0.2), we note that RL-each has a smaller RMSE than RL-meta, which exhibits a good coefficient estimation. One possible reason is that when the number of studies and informative covariates is very small, there no or little information can be borrowed to improve the estimation accuracy. As the number of studies and/or the number of informative variable tend to large, our RL-meta consistently has the best performance among the three methods including L-meta, RL-each, and L-each. This again verifies that borrowing information across similar studies can significantly improve parameter estimation and the accuracy of variable selection (Liu et al., 2011).

**Table 3:** Results for low-dimensional data with contamination.

	**(******n**, **p******) = (100, 50)**				
***M* = 2**		**RL-meta**	**L-meta**	**RL-each**	**L-each**
π = 0.2	Precision	0.635 (0.019)	0.248 (0.027)	0.338 (0.007)	**0.764** (0.032)
	Recall	**0.854** (0.016)	0.184 (0.019)	0.848 (0.010)	0.138 (0.011)
	*F*_1_	**0.728** (0.014)	0.362 (0.013)	0.480 (0.009)	0.301 (0.010)
	RMSE	0.376 (0.015)	0.449 (0.023)	**0.207** (0.009)	0.456 (0.017)
π = 0.5	Precision	0.664 (0.017)	0.537 (0.001)	0.374 (0.015)	**0.898** (0.018)
	Recall	**0.768** (0.026)	0.113 (0.026)	0.634 (0.023)	0.135 (0.010)
	*F*_1_	**0.701** (0.020)	0.252 (0.013)	0.465 (0.017)	0.243 (0.010)
	RMSE	**0.470** (0.021)	0.725 (0.024)	0.615 (0.019)	0.725 (0.026)
π = 0.9	Precision	0.715 (0.021)	0.340 (0.029)	0.391 (0.015)	**0.921** (0.012)
	Recall	**0.636** (0.030)	0.089 (0.008)	0.518 (0.021)	0.077 (0.005)
	*F*_1_	**0.658** (0.025)	0.188 (0.010)	0.445 (0.017)	0.159 (0.006)
	RMSE	**0.608** (0.027)	0.817 (0.031)	0.707 (0.026)	0.813 (0.034)
***M* = 8**		**RL-meta**	**L-meta**	**RL-each**	**L-each**
π = 0.2	Precision	**0.682** (0.009)	0.568 (0.043)	0.291 (0.005)	0.592 (0.032)
	Recall	**0.899** (0.010)	0.105(0.010)	0.842 (0.011)	0.051 (0.003)
	*F*_1_	**0.770** (0.012)	0.251 (0.005)	0.432 (0.006)	0.104 (0.004)
	RMSE	**0.216** (0.014)	0.289(0.021)	0.219 (0.018)	0.927 (0.013)
π = 0.5	Precision	0.691 (0.010)	0.128 (0.017)	0.358 (0.010)	**0.840** (0.018)
	Recall	**0.923** (0.011)	0.042 (0.005)	0.724 (0.012)	0.102 (0.003)
	*F*_1_	**0.787** (0.005)	0.118 (0.007)	0.479 (0.008)	0.181 (0.005)
	RMSE	**0.251** (0.016)	0.473 (0.021)	0.352 (0.019)	0.480 (0.023)
π = 0.9	Precision	0.828 (0.010)	0.238 (0.028)	0.358 (0.007)	**0.939** (0.011)
	Recall	**0.866** (0.010)	0.053 (0.006)	0.519 (0.012)	0.052 (0.002)
	*F*_1_	**0.839** (0.013)	0.161 (0.008)	0.423 (0.008)	0.098 (0.004)
	RMSE	**0.516** (0.027)	0.701(0.029)	0.680 (0.015)	0.699 (0.030)

*The presented values are the means of Precision, Recall, F_1_ score, and RMSE with standard errors in parentheses, respectively, averaged over 100 simulations. The bold values for Presicion, Recall, and F1 score are the highest values, and the bold value for RMSE is the lowest value*.

**Table 4:** Results for high-dimensional data with contamination data.

	**(******n**, **p******) = (150, 1, 000)**				
***M* = 2**		**RL-meta**	**L-meta**	**RL-each**	**L-each**
π = 0.2	Precision	0.510 (0.017)	0.076 (0.006)	**0.878** (0.009)	0.273 (0.043)
	Recall	**0.873** (0.009)	0.324 (0.014)	0.261 (0.005)	0.018 (0.005)
	*F*_1_	**0.613** (0.013)	0.125 (0.007)	0.202 (0.003)	0.246 (0.001)
	RMSE^*^	0.298 (0.032)	0.379 (0.045)	**0.161** (0.008)	0.316(0.032)
π = 0.5	Precision	**0.463** (0.024)	0.104 (0.009)	0.139 (0.006)	0.314 (0.044)
	Recall	**0.685** (0.027)	0.082 (0.004)	0.553 (0.020)	0.015 (0.003)
	*F*_1_	**0.563** (0.023)	0.082 (0.005)	0.226 (0.009)	0.136 (0.002)
	RMSE^*^	**0.387** (0.069)	0.548 (0.093)	0.425(0.060)	0.500 (0.073)
π = 0.9	Precision	0.414 (0.036)	0.029 (0.004)	0.108 (0.007)	**0.583** (0.046)
	Recall	**0.531** (0.030)	0.055 (0.006)	0.348 (0.023)	0.022 (0.003)
	*F*_1_	**0.499** (0.025)	0.070 (0.003)	0.181 (0.010)	0.107 (0.003)
	RMSE^*^	**0.435** (0.070)	0.548 (0.071)	0.489 (0.083)	0.561 (0.071)
***M* = 8**		**RL-meta**	**L-meta**	**RL-each**	**L-each**
π = 0.2	Precision	**0.660** (0.011)	0.053 (0.001)	0.120 (0.002)	0.374 (0.033)
	Recall	**0.915** (0.007)	0.664(0.009)	0.810 (0.008)	0.040 (0.004)
	*F*_1_	**0.760** (0.007)	0.097 (0.001)	0.208 (0.003)	0.106 (0.004)
	RMSE^*^	**0.210** (0.011)	0.374 (0.039)	0.227 (0.091)	0.292 (0.018)
π = 0.5	Precision	**0.660** (0.007)	0.044 (0.002)	0.145 (0.003)	0.383 (0.034)
	Recall	**0.906** (0.008)	0.323 (0.013)	0.675 (0.011)	0.019 (0.002)
	*F*_1_	**0.760** (0.005)	0.077 (0.003)	0.237 (0.005)	0.059 (0.003)
	RMSE^*^	**0.316** (0.098)	0.547 (0.107)	0.519 (0.095)	0.469 (0.026)
π = 0.9	Precision	**0.752** (0.011)	0.048 (0.002)	0.087 (0.002)	0.584 (0.024)
	Recall	**0.767** (0.021)	0.219 (0.011)	0.433 (0.010)	0.032 (0.002)
	*F*_1_	**0.753** (0.015)	0.080 (0.004)	0.144 (0.003)	0.062 (0.003)
	RMSE^*^	**0.358** (0.097)	0.614(0.085)	0.503 (0.082)	0.471 (0.074)

*The presented values are the means of Precision, Recall, F_1_ score, and RMSE with standard errors in parentheses, respectively, averaged over 100 simulations. The bold values for Presicion, Recall, and F1 score are the highest values, and the bold value for RMSE is the lowest value*.

## 4. Real Data Application

In this section, we consider three publicly available lung cancer datasets from GEO (https://www.ncbi.nlm.nih.gov/gds/). The first data are the gene expression signature of cigarette smoking (GSE10072), which contains the gene expression levels of 49 normal and 58 tumor tissues from 28 current smokers, 26 former smokers, and 20 never smokers, and each sample has 22,283 genes. The second data are the early stage non-small-cell lung cancer (GSE19188), which contains the gene expression levels of 65 adjacent normal and 91 tumor tissues, and each sample has 54,675 genes. The third data are the non-smoking female lung cancer in Taiwan (GSE19804), which contains the gene expression levels of 60 normal and 60 tumor tissues, and each sample has 54,675 genes. Consequently, there are 13,515 common genes shared between these three datasets. We map the probes of the raw data to gene names by annotation packages in Bioconductor. Also as per Hui et al. (2020) and Alfons et al. (2013), if multiple probes match a same gene, we compute the median values of these probes as the expression values for this gene and do the normalization for the raw expression data by the “affy” R package. Let |*t*_*kj*_| be the absolute value of standardized mean difference for the *j*th gene in the *k*th dataset and *T*_*j*_ = max(|*t*_1*j*_|, |*t*_2*j*_|, |*t*_3*j*_|). We select the 1,000 genes with the largest values of *T*_*j*_ for *j* = 1, …, 13, 515, and then perform the variable selection for the three datasets based on RL-meta, L-meta, RL-each, and L-each, respectively.

**Figure 6:**
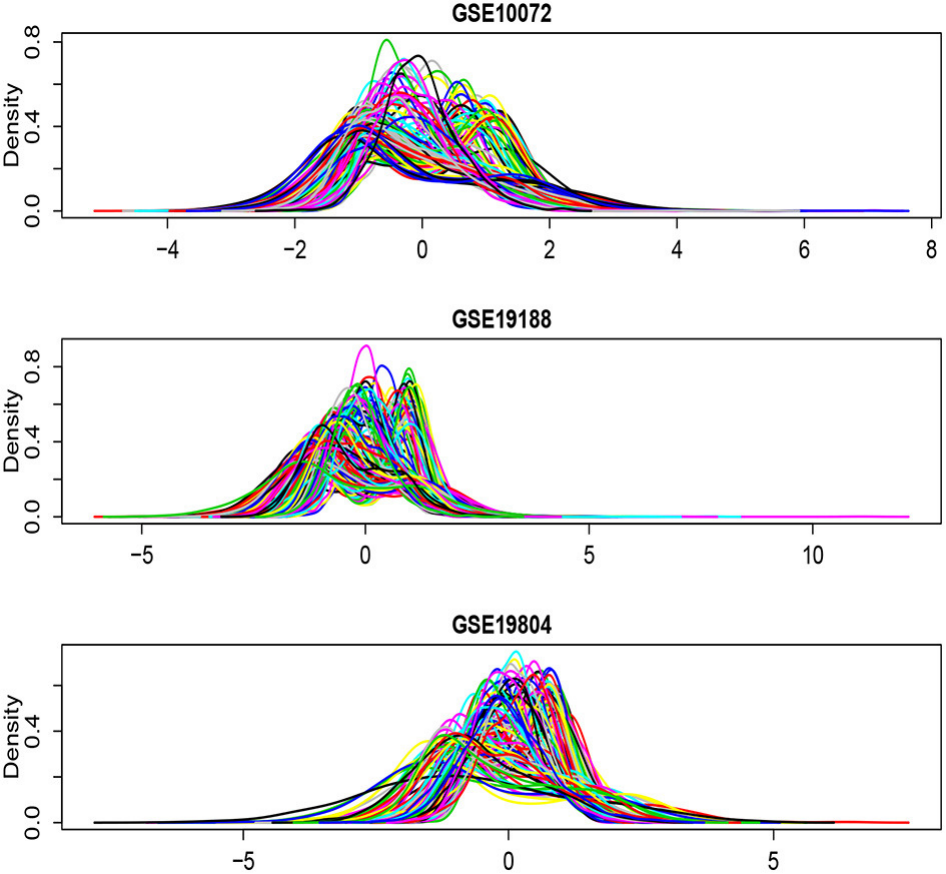
Density plots for each of the selected 1,000 genes in the GSE10072, GSE19198, and GSE19804, respectively.

Figure 6 shows the density plots for each of the selected 1,000 genes in the GSE10072, GSE19198, and GSE19804, respectively. The expression levels of some genes in GSE10072 and GSE19198 exhibit heavy-tailed distributions and may present outliers. Table 5 shows the detected informative genes by RL-meta, L-meta, RL-each, and L-each in the three lung cancer studies. We observe that RL-meta detects 7 more genes than L-meta, and both of the methods identify one common gene “COL10A1” between the three studies. In addition, RL-meta detects four overlapped informative genes in GSE19188 and GSE19804, whereas L-meta only detects 1 overlapped gene. Since GSE19188 and GSE19804 are both from the same Affymetrix Platform (U133 Plus 2.0), it is expected that RL-meta has a higher detection power and is also more reproducible than L-meta. Finally, RL-each and L-each detect 15 and 22 genes, respectively. Nevertheless, these two methods identify very different genes across the three studies and so may lack of reproducibility.

**Table 5:** Gene selections of RL-meta, L-meta, RL-each, and L-each in three lung cancer studies.

	**GSE10072**	**GSE19188**	**GSE19804**
RL-meta	**AF007147**	**AF007147**, ACSL4, GRIA1	
	**SVEP1**	**SVEP1**, ***EHD1**, **LGALSL***	***EHD1**, **LGALSL***
	COL10A1	COL10A1, ***FUT2***	COL10A1, ***FUT2***
L-meta	COL10A1	ACSL4, COL10A1	COL10A1
	**AF007147**	**AF007147**	FUT2
	**LINC01140**	**LINC01140**	
RL-each	CA4, CD36	AGER, CA4	CA4, SGCG
	SPP1, GPM6A	GDF10, GAPDH	MME
	FAM107A, FCN3	FAM189A2, LRRC36	
L-each	PDE2A	AOX1, AF007147, ACADL	ALDH18A1, COL10A1
	SPP1	GAPDH, PAFAH1B3	GOLM1, MME
	TNXA	LRRC36, LINC00341	EFNA4, SORD
		HIST1H2BD, PPBP	SPOCK2, HN1L
		CCL23, FCN3	

*The bolded and italic bolded names are overlapped identified genes between GSE10072 and GSE19188 and between GSE19188 and GSE19804, respectively. The underlined names are overlapped identified genes across the three studies*.

To further compare the performance of the four methods, we also consider to create outliers for the three datasets. Specifically, we select the first eight samples from each of the three datasets, and then add a number 10 to the expression levels of those informative genes. In order to generate outliers instead of leverage points, we assign the labels of those samples to their opposite class. Table 6 shows the identified informative genes with RL-meta, L-meta, RL-each, and L-each in the artificially created three datasets. L-meta and L-each both identify quite different genes between the artificially created datasets and the original datasets, whereas RL-meta and RL-each identify more common genes between the artificial created datasets and the original datasets. In addition, RL-meta detects four overlapped informative genes in artificially created GSE19188 and GSE19804, whereas L-meta only detects one overlapped gene. As we discussed in the analysis of original datasets, GSE19188 and GSE19804 are both from the same Affymetrix Platform, and hence it is expected that RL-meta is more reproducible than L-meta. To conclude, RL-meta is more robust and tends to be more powerful when outliers present in the datasets.

**Table 6:** Gcta, L-meta, RL-each, and L-each in three lung cancer studies.

	**GSE10072**	**GSE19188**	**GSE19804**
RL-meta	COL10A1	COL10A1, ACSL4	COL10A1
	SPOCK2	***FUT2***, GRIA1, TYRP1	***FUT2***
	LINC01140	***EHD1***, AF007147	***EHD1***
L-meta	MIF	***CFP***, MIF	***CFP***, MIF, GOLM1
	KCNJ8	KCNJ8	KCNJ8, COL10A1
RL-each	CA4	AGER, CA4	CA4, SGCG
	GPM6A	LRRC36, GDF10	SH3GL3, MASP1
			COL10A1, SPP1
L-each	FAM107A	AGER, GAPDH	COL10A1
	SPP1	PAFAH1B3, NEK2	GOLM1
		MIF, HIST1H2BD	SPOCK2

*The bolded and italic bolded names are overlapped identified genes between GSE10072 and GSE19188 and between GSE19188 and GSE19804, respectively. The underlined names are overlapped identified genes across the three studies*.

## 5. Discussion

In this paper, we propose a robust method for meta-analyzing multiple studies with high-dimensional data. Our method is based on a two-step procedure including a search step for a clean subset from each study and a reweighting scheme to improve the estimation efficiency. In particular, we incorporate the bi-level variable selection technique into both of the two steps. Our new robust method has the capacity to capture the sparsity at both the study and group levels so as to better integrating the structural information that can enhance the parameter estimation and variable selection. Simulation studies demonstrate that, in the presence of outliers, our proposed method can provide better parameter estimation and also identify informative covariates more accurately than the existing strategies, especially when the dimension is large. We also provide a comparison of computational cost for RL-meta, RL-each, L-meta, and L-each in Table A1. We note that RL-meta and RL-each suffer from a heavy computational burden. The main reason is that the two robust methods need to perform *C*-steps with different starting subsets, and hence the number of iterations is considerably higher than the classical lasso-based methods.

In addition, the lasso-based variable selection methods usually suffer from a low power when some covariates are highly correlated. As an alternative, Zou and Hastie (2005) and Tibshirani et al. (2005) proposed the elastic net and the fused lasso penalty to handle correlations among covariates. Following this direction, our RL-meta may further be improved by incorporating the correlated covariates. Specifically, with the hierarchical reparameterization in Theorem 1, one possible extension of (2.4) can be given as:

$$\begin{array}{l} Q_{net}\left(H,\alpha ,\gamma \right)=\overset{M}{\underset{k=1}{\sum }}\underset{i\in H_{k}}{\sum }d\left(x_{ki}\beta_{k},y_{ki}\right)+\overset{p}{\underset{j=1}{\sum }}\left(|\alpha_{j}|+|\alpha_{j}{|}^{2}\right)+\lambda_{1}\overset{M}{\underset{k=1}{\sum }}\\ \;\overset{p}{\underset{j=1}{\sum }}\left(|\gamma_{kj}|+|\gamma_{kj}{|}^{2}\right). \end{array}$$

We leave this problem for further theoretical and numerical studies.

Finally, we note that Bayesian meta-analysis is also a popular approach for the integration of multiple studies. Recently, Cai et al. (2020) proposed a Bayesian variable selection approach for joint modeling multiple datasets. They developed a hierarchical spike-and-slab prior (a Bayesian version of the bi-level lasso penalty) to borrow information across the studies, which is shown to have a higher power for detecting informative single nucleotide polymorphisms in genome-wide association studies (GWAS). In addition, Pickrell (2014) proposed a Bayesian hierarchical model for GWAS data by borrowing information from functional genomic studies. As a future work, it would be worthwhile to develop such Bayesian methods for robustly meta-analyzing multiple datasets and make a comparison with the RL-meta and L-meta methods introduced in the current paper.

## Data Availability Statement

Publicly available datasets were analyzed in this study. This data can be downloaded from the link: https://www.ncbi.nlm.nih.gov/gds/.

## Author Contributions

ZH developed the study and performed the simulation studies and the real data analysis. YZ and TT initiated the study and provided helpful discussions. All authors contributed to the article and approved the final version.

## Conflict of Interest

The authors declare that the research was conducted in the absence of any commercial or financial relationships that could be construed as a potential conflict of interest.
