# Supplementary material for: Meta-Analyzing Multiple Omics Data With Robust Variable Selection
Source: Front Genet. 2021 Jul 5;12:656826. doi: 10.3389/fgene.2021.656826 (PMC8288516; doi:10.3389/fgene.2021.656826)
Supplement: Supplementary file 1 [file Presentation_1.pdf]

Supplementary Material

APPENDIX A: PROOF OF THE THEOREMS

Before proving the two theorems, we first define the function $\phi(x)$ as in Bianco and Yohai (1996) and Crous and Haesbroeck (2003).

DEFINITION A1. *Let* $F(x) = 1/(1+\exp(-x))$, $U(x) = \int_0^x \rho'(-\ln(t))dt$ *for* $0 \le x \le 1$, *and*

$$\rho(x) = \begin{cases} x\exp(-\sqrt{c}), & x \le c, \\ -2\exp(-\sqrt{x})(1+\sqrt{x}) + \exp(-\sqrt{x})(2(1+\sqrt{c})+c), & \text{otherwise.} \end{cases}$$

If we let $c = 0.5$, *then the function* $\phi(x)$ *is defined on* $x \in R$ *and can be expressed as*

$$\phi(x) = \rho(-\ln(1-F(x))) + U(F(x)) + U(1-F(x)) - U(1).$$

A.1 Proof of Theorem 1

We follow the similar idea as in the proof of He et al. (2016). Let $\boldsymbol{g} = (g_1, \ldots, g_p)^T$ with $g_j \ge 0$ for all $j = 1, \ldots, p$. Then by the Cauchy-Schwartz inequality,

$$\begin{aligned} Q_2(\mathcal{H}, \boldsymbol{g}, \boldsymbol{\beta}) &= \sum_{k=1}^{M}\sum_{i\in H_k} d(\boldsymbol{x}_{ki}^T\boldsymbol{\beta}_k, y_{ki}) + \sum_{j=1}^{p} g_j + \frac{\lambda_2}{4}\sum_{j=1}^{p} g_j^{-1}\left(\sum_{k=1}^{M}|\beta_{kj}|\right), \\ &\ge \sum_{k=1}^{M}\sum_{i\in H_k} d(\boldsymbol{x}_{ki}^T\boldsymbol{\beta}_k, y_{ki}) + \sum_{j=1}^{p}\left(\lambda_2\sum_{k=1}^{M}|\beta_{kj}|\right)^{1/2}, \end{aligned}$$

where the equality holds if and only if $g_j = \lambda_2^{1/2}(\sum_{k=1}^M |\beta_{kj}|)^{1/2}$. Consequently, if $\widehat{\boldsymbol{\beta}}_{\mathcal{H}} = (\widehat{\boldsymbol{\beta}}_{1,\mathcal{H}}^T, \ldots, \widehat{\boldsymbol{\beta}}_{M,\mathcal{H}}^T)^T$ is a solution for the minimization of (2.2), then by letting $\lambda = \lambda_2^{1/2}$ and $\hat{g}_j = \lambda_2^{1/2}(\sum_{k=1}^M |\hat{\beta}_{kj}|)^{1/2}$, $(\widehat{\boldsymbol{\beta}}_{\mathcal{H}}, \hat{g}_1, \ldots, \hat{g}_p)$ is also a solution for the minimization of $Q_2(\mathcal{H}, \boldsymbol{\alpha}, \boldsymbol{\gamma})$. This indicates that the minimization problem of $Q_2(\mathcal{H}, \boldsymbol{g}, \boldsymbol{\beta})$ is equivalent to that for $Q(\mathcal{H}, \boldsymbol{\beta})$.

Let also $\alpha_j = g_j$, $\gamma_{kj} = g_j^{-1}\beta_{kj}$ for $g_j \ne 0$ and $\gamma_{kj} = 0$ for $g_j = 0$. We have

$$\begin{aligned} Q_2(\mathcal{H}, \boldsymbol{\alpha}, \boldsymbol{\gamma}) &= \sum_{k=1}^{M}\sum_{i\in H_k} d(\boldsymbol{x}_{ki}^T\boldsymbol{\beta}_k, y_{ki}) + \sum_{j=1}^{p}|\alpha_j| + \frac{\lambda^2}{4}\sum_{j=1}^{p}\left(\sum_{k=1}^{M}|\gamma_{kj}|\right) \\ &= Q_1(\mathcal{H}, \boldsymbol{\alpha}, \boldsymbol{\gamma}). \end{aligned}$$

This shows that the minimization problems for the objective functions (2.2) and (2.4) are equivalent to each other.

A.2 Proof of Theorem 2

By the construction of the approximate clean subset, we have

$$Q(\mathcal{H}_{t+1}, \widehat{\boldsymbol{\beta}}_{\mathcal{H}_t}) \le Q(\mathcal{H}_t, \widehat{\boldsymbol{\beta}}_{\mathcal{H}_t}).$$

Noting also that $Q(\mathcal{H}_{t+1}, \widehat{\boldsymbol{\beta}}_{\mathcal{H}_{t+1}})$ is the minimum value with respect to $\boldsymbol{\beta}$, we further have

$$Q(\mathcal{H}_{t+1}, \widehat{\boldsymbol{\beta}}_{\mathcal{H}_{t+1}}) \le Q(\mathcal{H}_{t+1}, \widehat{\boldsymbol{\beta}}_{\mathcal{H}_t}).$$

This leads to $Q(\mathcal{H}_{t+1}, \widehat{\boldsymbol{\beta}}_{\mathcal{H}_{t+1}}) \le Q(\mathcal{H}_t, \widehat{\boldsymbol{\beta}}_{\mathcal{H}_t})$.

APPENDIX B: ADDITIONAL SIMULATION RESULTS

B.1 The plots of coefficient estimates for L-each and RL-each

Figures S1-S4 show the average values of the estimates for each coefficient with the confidence intervals (mean $\pm 3\times$ standard error) for $M = 2$ studies. The parameter estimation is based on L-each and RL-each with clean and contamination data, respectively.

B.2 A comparison for time consumption

Table S1 summarizes the computational time of our RL-meta and the other three methods adopted in Section 3. The time (in seconds) for one simulation is reported using the R software with Intel Core (TM) 3.20GH processor. We note that RL-meta and RL-each have a larger computational burden than L-meta and L-each. This may due to that the two robust methods need a C-step to search for an outlier-free subset iteratively with different initial subsets.

Table S1. The time consumption (in seconds) of RL-meta, L-meta, RL-each and L-each with $(M, n, p, \pi) = (2, 150, 1000, 0.5)$, respectively.

	RL-meta	RL-each	L-meta	L-each
Run Time	674.06	447	1.64	1.19

REFERENCES

Bianco, A. M. and Yohai, V. J. (1996). *Robust Estimation in the Logistic Regression Model* (New York: Springer)

Crous, C. and Haesbroeck, G. (2003). Implementing the Bianco and Yohai estimator for logistic regression. *Computational Statistics and Data Analysis* 44, 273–295

He, Q., Zhang, H. H., Avery, C. L., and Lin, D. Y. (2016). Sparse meta-analysis with high-dimensional data. *Biostatistics* 2, 205–220

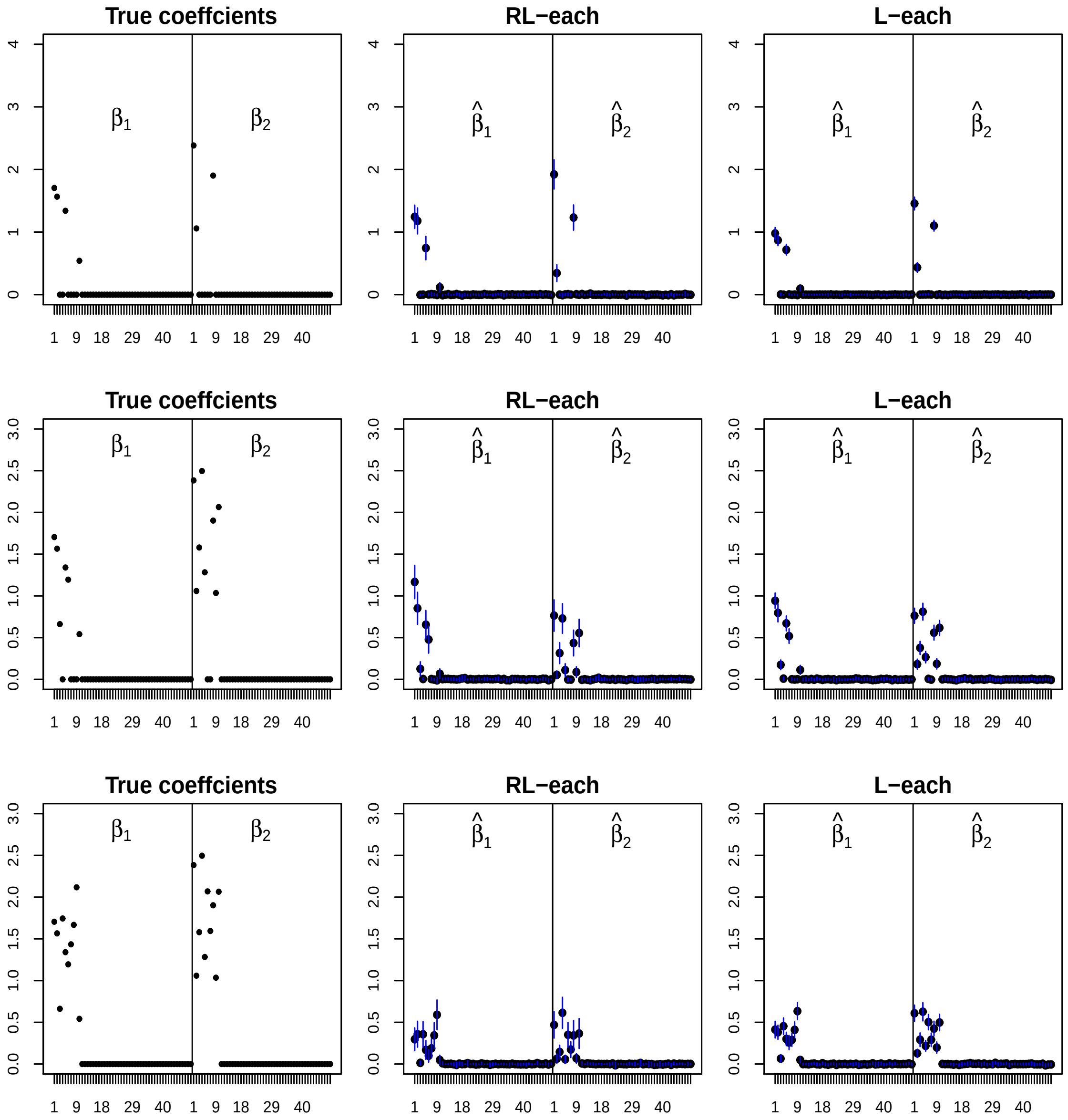

Figure S1. The coefficient estimates with clean data for $M = 2$ and $(n, p) = (100, 50)$. The blue points and lines represent the estimated values and the interval estimates of coefficients over 100 simulations. Rows from top to bottom correspond to $\pi_0 = 0.2, 0.5, 0.9$, respectively.

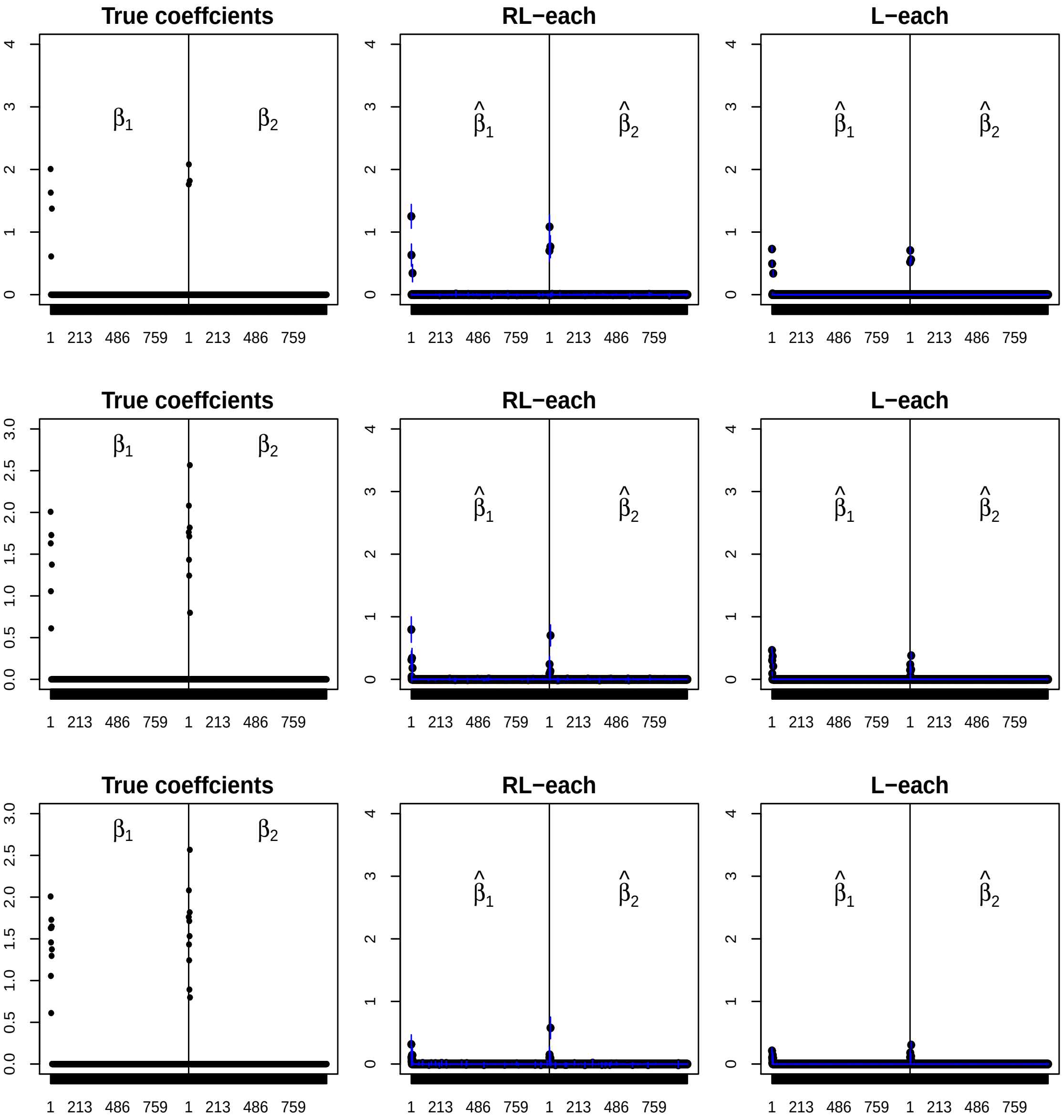

Figure S2. The coefficient estimates with clean data for $M = 2$ and $(n, p) = (150, 1000)$. The blue points and lines represent the estimated values and the interval estimates of coefficients over 100 simulations. Rows from top to bottom correspond to $\pi = 0.2, 0.5, 0.9$, respectively.

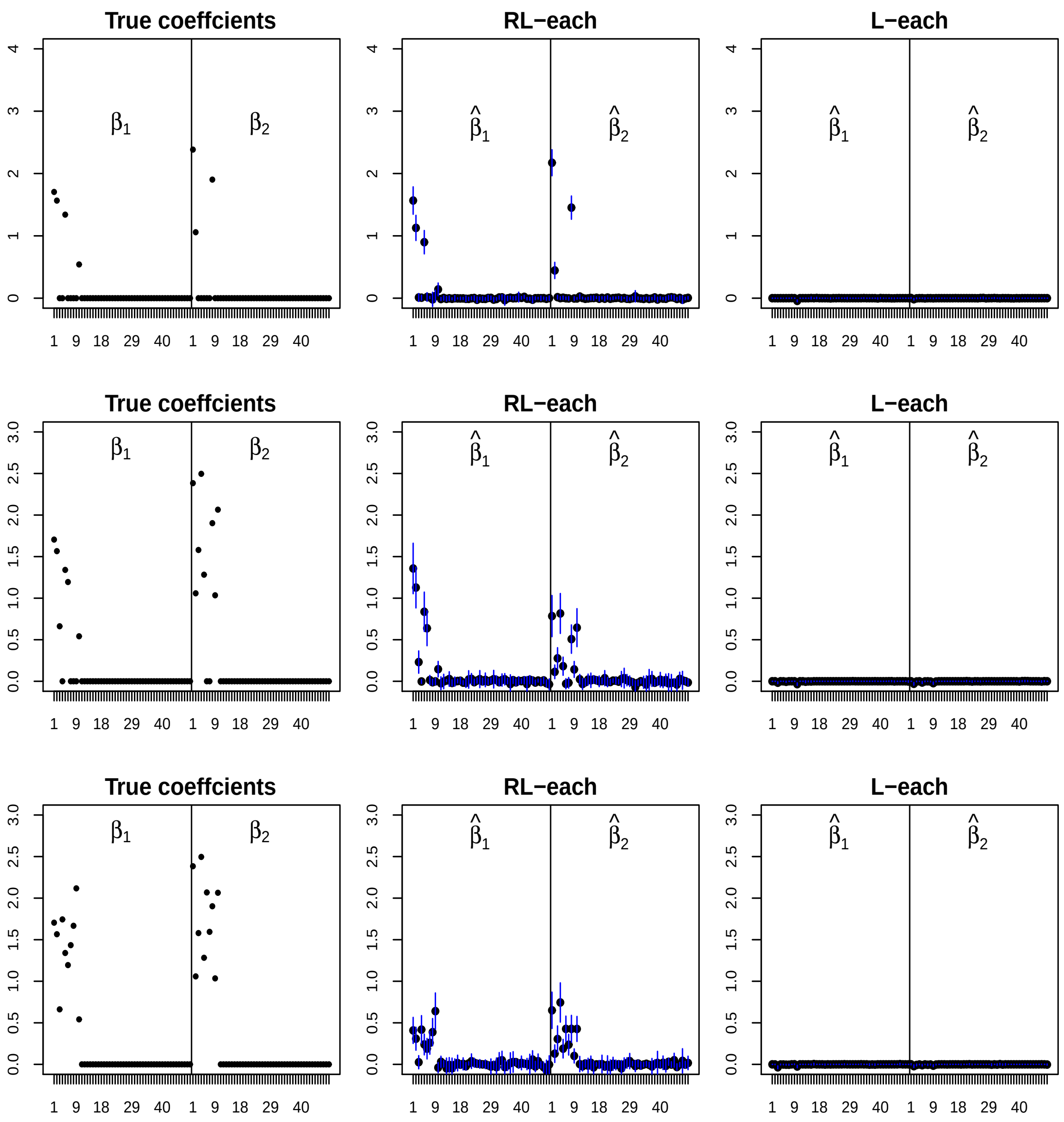

Figure S3. The coefficient estimates with contamination data for $M = 2$ and $(n, p) = (100, 50)$. The blue points and lines represent the estimated values and the interval estimates of coefficients over 100 simulations. Rows from top to bottom correspond to $\pi_0 = 0.2, 0.5, 0.9$, respectively.

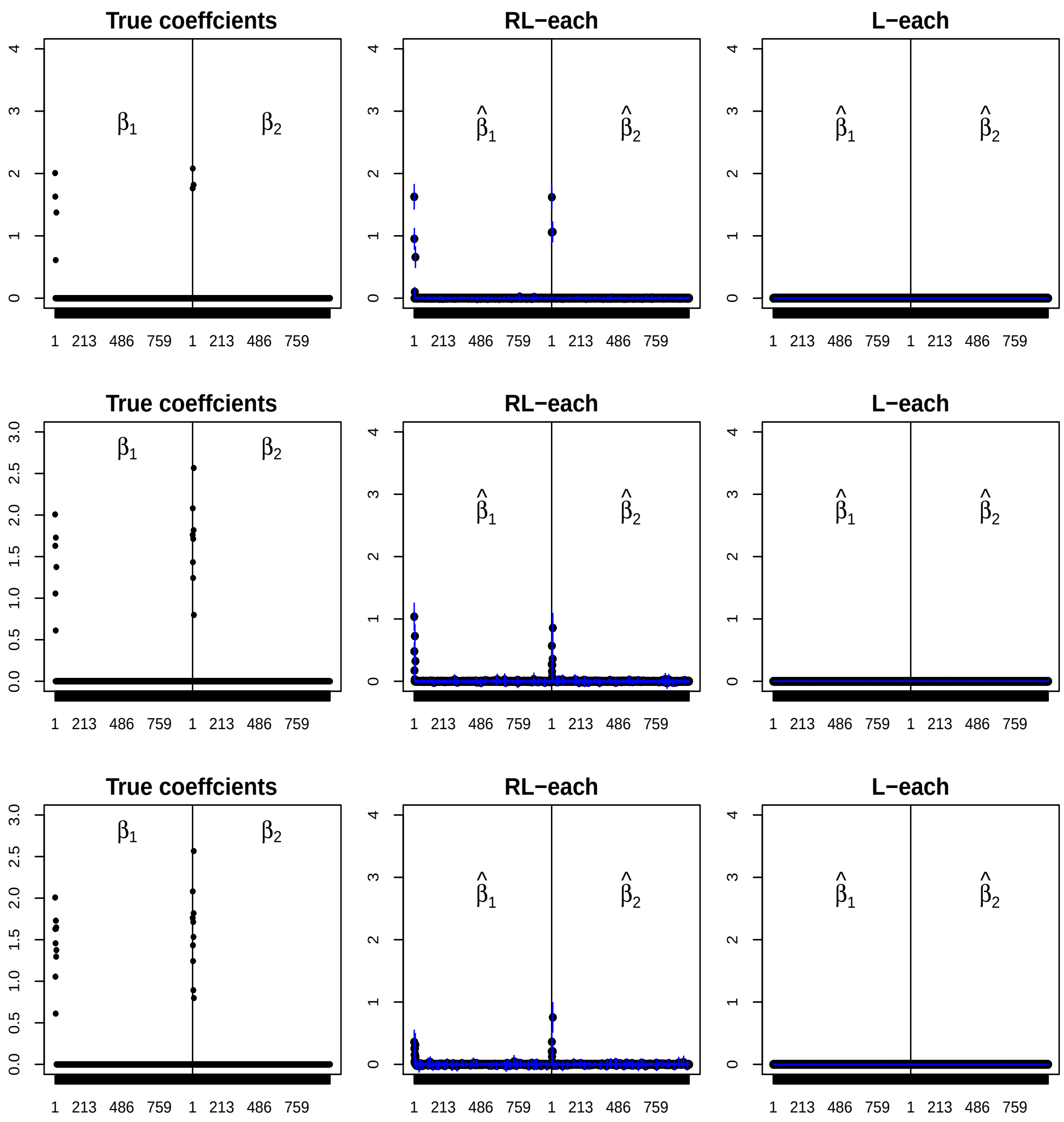

Figure S4. The coefficient estimates with contamination data for $M = 2$ and $(n, p) = (150, 1000)$. The blue points and lines represent the estimated values and the interval estimates of coefficients over 100 simulations. Rows from top to bottom correspond to $\pi = 0.2, 0.5, 0.9$, respectively.
